# Anti-dense fine speckled 70 (DFS70) autoantibodies: correlates and increasing prevalence in the United States

**DOI:** 10.3389/fimmu.2023.1186439

**Published:** 2023-06-23

**Authors:** Gregg E. Dinse, Bing Zheng, Caroll A. Co, Christine G. Parks, Clarice R. Weinberg, Frederick W. Miller, Edward K. L. Chan

**Affiliations:** ^1^ Public Health and Scientific Research, Social and Scientific Systems, Inc., a DLH Holdings Corp. Company, Durham, NC, United States; ^2^ Department of Oral Biology, University of Florida, Gainesville, FL, United States; ^3^ Department of Laboratory Medicine, Renji Hospital, School of Medicine, Shanghai Jiao Tong University, Shanghai, China; ^4^ Epidemiology Branch, National Institute of Environmental Health Sciences, National Institutes of Health, Research Triangle Park, NC, United States; ^5^ Biostatistics and Computational Biology Branch, National Institute of Environmental Health Sciences, National Institutes of Health, Research Triangle Park, NC, United States; ^6^ Clinical Research Branch, National Institute of Environmental Health Sciences, National Institutes of Health, Research Triangle Park, NC, United States

**Keywords:** antinuclear antibodies (ANA), autoantibody, autoimmune disease, dense fine speckled (DFS), dense fine speckled 70 (DFS70), National Health and Nutrition Examination Survey (NHANES), population study

## Abstract

**Objective:**

Recent studies report high-titer anti-dense fine speckled 70 (DFS70) autoantibodies in persons with inflammatory conditions, but the clinical significance remains unclear. Our goals were to estimate anti-DFS70 autoantibody prevalence, identify correlates, and assess time trends.

**Methods:**

Serum antinuclear antibodies (ANA) were measured by indirect immunofluorescence assay on HEp-2 cells in 13,519 participants ≥12 years old from three time periods (1988–1991, 1999–2004, 2011–2012) of the National Health and Nutrition Examination Survey. ANA-positive participants with dense fine speckled staining were evaluated for anti-DFS70 antibodies by enzyme-linked immunosorbent assay. We used logistic models adjusted for survey-design variables to estimate period-specific anti-DFS70 antibody prevalence in the US, and we further adjusted for sex, age, and race/ethnicity to identify correlates and assess time trends.

**Results:**

Women were more likely than men (odds ratio (OR)=2.97), black persons were less likely than white persons (OR=0.60), and active smokers were less likely than nonsmokers (OR=0.28) to have anti-DFS70 antibodies. The prevalence of anti-DFS70 antibodies increased from 1.6% in 1988-1991 to 2.5% in 1999-2004 to 4.0% in 2011-2012, which corresponds to 3.2 million, 5.8 million, and 10.4 million seropositive individuals, respectively. This increasing time trend in the US population (P<0.0001) was modified in some subgroups and was not explained by concurrent changes in tobacco smoke exposure. Some, but not all, anti-DFS70 antibody correlates and time trends resembled those reported for total ANA.

**Conclusion:**

More research is needed to elucidate anti-DFS70 antibody triggers, their pathologic or potentially protective influences on disease, and their possible clinical implications.

## Introduction

Autoimmune diseases are a diverse group of disorders characterized by damaging immune responses to self-antigens, and which, outside of some environmental and iatrogenic exposures, are of unknown etiology (1, 2). They impact 5% or more of the population, with increasing rates observed recently (3–8), but it remains unclear whether these positive time trends are due to changes in recognition and diagnosis, or if they represent true temporal increases in incidence, possibly related to environmental exposures (9).

Antinuclear antibodies (ANA) are a common biomarker of autoimmunity, observed in patients with various autoimmune diseases. However, ANA are also observed in the general population and are associated with sociodemographic factors such as older age and female sex (10, 11), genetic factors (12), and environmental exposures, including infections, medications, and organic and inorganic chemicals and toxins (4, 13, 14). Parallel to increases in autoimmune diseases, ANA prevalence has also increased (15).

There are many types and target specificities of ANA, and some are more clinically relevant than others (16, 17). The dense fine speckled (DFS) staining pattern observed by indirect immunofluorescence assay (IFA) on HEp-2 cells is often, but not always, indicative of autoantibodies to a specific 70 kDa protein known as DFS70 (18, 19). The first reports on anti-DFS70 antibodies suggested associations with atopic dermatitis and other conditions (20). More recent studies suggest their association with younger age groups (21–23) and indicate they are the second most common IFA staining pattern in apparently healthy individuals (16). The detection of high-titer monospecific anti-DFS70 antibodies can help rule out the diagnosis of systemic autoimmune rheumatic diseases (SARD), as they are rarely observed in persons with these conditions (16, 17, 24–27).

Our primarily exploratory and descriptive study focused on anti-DFS70 antibodies and used US National Health and Nutrition Examination Survey (NHANES) data from 1988 through 2012 to estimate prevalence, identify correlates, and assess time trends (15). In addition to evaluating anti-DFS70 antibody associations with standard sociodemographic factors such as sex, age, race/ethnicity, education, and economic status, we also evaluated several health-related factors, including body mass index, tobacco smoke exposure, alcohol consumption, C-reactive protein level, general health status, poor health preventing work, and allergens. As a secondary goal, we investigated whether time trends in factor-based subgroups differed from the overall time trend, which might suggest trend modification. Additionally, we investigated whether anti-DFS70 antibody correlates and time trends resembled those observed previously for the broader category of total ANA (10, 15), or alternatively might have either driven or attenuated them.

## Subjects and methods

### Study population

We measured ANA in 13,519 NHANES participants ≥12 years old, sampled from three time periods: 1988-1991 (4,727 persons), 1999-2004 (4,527 persons), and 2011-2012 (4,265 persons) as detailed previously (15). The NHANES sampled the civilian noninstitutionalized US population and provided sampling weights to adjust for nonresponse and the probability of selection into each ANA subsample (28), which allows for nationally representative estimates. The NHANES protocol was approved by the Human Subjects Institutional Review Board of the US Centers for Disease Control and Prevention (CDC). Written informed consent was obtained from all participants.

### Antibody assessment

Surplus NHANES serum samples from the three cohorts were collected at the same point in time and ANA were assessed by IFA on HEp-2 cells with a NOVA View system (INOVA Diagnostics, San Diego, CA) and graded 0-4 as described earlier (10, 15). Nuclear, cytoplasmic, and mitotic patterns of ANA were identified according to international consensus criteria (17). Independent readings were initially made by two experienced evaluators, blinded with respect to sample characteristics and time period. The two raters agreed on >95% of the ANA intensities and patterns; differences were resolved by consensus or adjudicated by a third blinded rater (EKLC). Repeat testing of random samples showed >98% concordance (10). All ANA-positive participants with the DFS (AC-2) IFA staining pattern were evaluated by an enzyme-linked immunosorbent assay (ELISA) for anti-DFS70 antibodies (Medical & Biological Laboratories, 7808, Japan) and classified as positive when the average value in two duplicate wells was >15 unit/mL, per manufacturer instructions. All sera were analyzed using the same methods, same reagents, and same equipment by the same evaluators in the same laboratory. Twenty-three randomly selected anti-DFS70-positive sera and all sera with a value near 15 were repeated and validated using the same assay methodology.

### Endpoints

Our statistical analysis focused on binary indicators of total ANA and anti-DFS70 antibody positivity/negativity. Participants with ANA grades 1-4 were treated as positive, while those with a grade of 0 were treated as negative (15). Among ANA-positive participants with DFS (AC-2) IFA staining, those with anti-DFS70 antibodies detected by ELISA were treated as positive, while all others were treated as negative, including those not tested with ELISA because they were ANA-negative. Our primary goal was to estimate prevalence, identify correlates, and assess time trends for anti-DFS70 antibodies, but for completeness we did the same for total ANA. We investigated the degree to which anti-DFS70 antibodies affected the total ANA results with a sensitivity analysis of “other” ANA, which excluded persons positive for anti-DFS70 antibodies and treated the remaining ANA-positives as positive for other ANA.

### Explanatory variables

Sex, age, and race/ethnicity are known correlates of ANA and modifiers of ANA time trends (15), so we included these variables in our anti-DFS70 antibody analyses. Age was treated as a continuous variable when used as an adjustment covariate but categorized into three groups when used for stratification: adolescents (12-19 years), younger adults (20-49 years), or older adults (≥50 years). Race/ethnicity was categorized as white persons, black persons, Mexican Americans, or others. We also examined body mass index (BMI), poverty income ratio (PIR), current smoking exposure, alcohol consumption, education, and C-reactive protein, which were included and defined in our previous ANA studies (10). For exploratory purposes, we investigated health-related variables related to general health status, poor health preventing work, and allergens. Self-reported general health status was categorized as excellent, very good, good, or fair/poor. An indicator that poor health prevented work was based on whether participants reported that physical, mental, or emotional problems kept them from working. An allergen indicator was based on whether a positive skin test, administered by NHANES staff at enrollment, showed a participant was allergic to Alternaria alternata, Bermuda grass, cats, German cockroaches, dust mites, peanuts, short ragweed, Russian thistle, rye grass, or white oak. **Supplemental Table S1** lists all participant characteristics studied.

### Data availability

There were 13,519 NHANES participants in our study. Data on sex, age, and race/ethnicity were available for everyone. However, for some participants, data on the other variables were not collected, were purposely excluded, or were missing. For example, general health status was not collected by NHANES for any participants in Period 1; was only collected for adults in some survey cycles, so we excluded all adolescents (ages <20 years) for consistency in the analysis; and was missing for some participants in Periods 2 and 3. For each variable and every period (and all periods combined), **Supplemental Table S2** lists the number and percentage of participants who were analyzed, were not surveyed, were excluded from the analysis, and were missing data; several NHANES restrictions based on age and period (or survey cycle) are described in the footnotes.

### Statistical analysis

We estimated the prevalence (population proportion) of total ANA, anti-DFS70 antibodies, and other ANA, both overall and in factor-based subgroups. Prevalence estimates and 95% confidence intervals (CIs) were derived from logistic regression models for antibody positivity, adjusted for the survey design variables (sampling strata, clusters, and weights), the factor defining the subgroup, and in some cases time period. The number of antibody-positive persons in each period was estimated by multiplying our period-specific prevalence estimate by the period-specific CDC estimate of the civilian noninstitutionalized US population ≥12 years old (https://wwwn.cdc.gov/nchs/nhanes/ResponseRates.aspx#population-totals). We evaluated the association between antibody status and an individual factor category, relative to a reference category, with a prevalence odds ratio (OR) and a 95% CI from a logistic model for antibody positivity, adjusted for period, sex, age, and race/ethnicity. The association between antibody status and a factor in its entirety was assessed by an F-test from a statistical contrast. We investigated time trends overall and in subgroups (to identify trend modifiers) by fitting two logistic models for antibody positivity; both adjusted for sex, age, and race/ethnicity. One model added a categorical covariate for period, from which ORs and CIs were calculated to assess how antibody positivity differed in each period relative to the first. The other model added a quantitative covariate for the time between period midpoints (0, 12, or 22 years), and a time trend was assessed with a χ^2^-test to judge whether the period covariate’s coefficient was zero. We assessed whether the time trends in all subgroups defined by a given factor truly differed or simply reflected the overall trend (e.g., were male and female trends distinct, such that sex was a trend modifier) by adding a time-by-factor interaction to the model and testing whether its coefficient(s) equaled zero. Because smoking habits changed over these same time periods, we also explored whether time trends were attenuated after further adjustment for smoking exposure (i.e., could trends be explained by those behavioral changes).

All analyses were conducted using SAS version 9.4 (SAS Institute, Cary, NC) and all accounted for the survey design variables (sampling strata, clusters, and weights). The sampling weights allowed for population-representative estimates by adjusting for survey nonresponse and selection probabilities (28). The covariate-adjusted analyses incorporated a restricted cubic spline (29) in age, categorical variables for sex and race/ethnicity, and either a categorical or quantitative variable for period. We used the SurveyLogistic procedure to perform the logistic regression analyses, with domain statements to properly handle the sampling weights in subgroup analyses. Variance estimates, used to construct the 95% CIs, were obtained by the Taylor series method. All P-values were 2-sided and unadjusted for multiple comparisons.

## Results

### Participant characteristics

The sample distributions of all participant characteristics considered in our analyses are shown in **Supplemental Table S1**, for each time period separately and for all periods combined. The proportions of some characteristics were relatively consistent across periods, while others varied. Sample distributions can be useful, but these unadjusted proportions are not nationally representative estimates because they do not account for the survey-sampling weights.

### Antibody positivity

Of the 13,519 participants, 1,857 (13.7%) were positive for total ANA and 312 (2.3%) were positive for anti-DFS70 antibodies. These counts and percentages are broken down into subgroups defined by categories of selected factors in **Table 1** (and additional factors in **Supplemental Table S3**). Though not shown, 484 (3.6%) of the 13,519 participants had the DFS (AC-2) staining pattern, of which 312 (64.5%) were positive for anti-DFS70 antibodies. Note that these unadjusted antibody positivity proportions are not nationally representative prevalence estimates.

**Table 1:** Sample distributions of total ANA and anti-DFS70 antibody positivity overall and in selected subgroups.

Factor of lnterest	Subgroup	Number Analyzed ^a^	Number (%) of Participants Positive for:	
			Total ANA	Anti-DFS70
Overall	All participants	13,519	1,857 (13.7)	312 (2.3)
Time Period	1988-1991	4,727	643 (13.6)	77 (1.6)
	1999-2004	4,527	545 (12.0)	101 (2.2)
	2011-2012	4,265	669 (15.7)	134 (3.1)
Sex	Males	6,641	613 (9.2)	75 (1.1)
	Females	6,878	1,244 (18.1)	237 (3.5)
Age (years)	Adolescents (12-19)	2,541	234 (9.2)	60 (2.4)
	Younger adults (20-49)	5,853	663 (11.3)	147 (2.5)
	Older adults (≥50)	5, 125	960 (18.7)	105 (2.1)
Race/Ethnicity	White persons	5,686	748 (13.2)	143 (2.5)
	Black persons	3, 123	488 (15.6)	58 (1.9)
	Mexican Americans	3,016	394 (13.1)	73 (2.4)
	Others	1,694	227 (13.4)	38 (2.2)
Body Mass Index (BMI)	Underweight/healthy	5,524	734 (13.3)	124 (2.2)
	Overweight	4, 102	552 (13.5)	90 (2.2)
	Obese	3,797	551 (14.5)	95 (2.5)
Poverty Income Ratio (PIR)	At/above poverty (≥l)	9,405	1,321 (14.1)	236 (2.5)
	Below poverty (<l)	2,943	368 (12.5)	53 (1.8)
Current Smoking Exposure (ng/ml of cotinine)	None (<0.05)	4,624	747 (16.2)	147 (3.2)
	Secondhand (0.05-15)	5,481	695 (12.7)	130 (2.4)
	Active (>15)	3, 183	374 (11.8)	31 (1.0)
Alcohol Consumption (age ≥20 years)	None	3,834	666 (17.4)	107 (2.8)
	Light	3,215	457 (14.2)	73 (2.3)
	Moderate/heavy	2,378	240 (10.1)	38 (1.6)

ANA, antinuclear antibodies; Anti-DFS70, subclass of ANA that had the DFS staining pattern and bound the 70 kDa DFS protein in an enzyme-linked immunosorbent assay; DFS, dense fine speckled.

^a^ For some factors, the subgroup counts do not sum to the overall total of 13,519 because some participants were not surveyed, were excluded, or were missing data (see Supplemental Table S2 for details and exact counts).

### Prevalence estimates

Adjusted only for the survey-design variables, the period-specific population-representative estimates of anti-DFS70 antibody prevalence are 1.57% (95% CI=1.20-2.04%) in 1988-1991, 2.48% (95% CI=1.85-3.33%) in 1999-2004, and 4.04% (95% CI=3.12-5.23%) in 2011-2012 (**Table 2**). The corresponding estimated numbers of individuals in the US who were positive for anti-DFS70 antibodies are 3.2 million (95% CI=2.4-4.1), 5.8 million (95% CI=4.3-7.8), and 10.4 million (95% CI=8.0-13.5). For comparison, the respective period-specific prevalence estimates for total ANA are 11.0% (95% CI=9.7-12.5%), 11.4% (95% CI=10.2-12.8%), and 16.1% (95% CI=14.5-17.9%), and the estimated numbers of ANA-positive individuals are 22.4 million (95% CI=19.6-25.4), 26.6 million (95% CI=23.8-29.7), and 41.6 million (95% CI=37.4-46.1). Prevalence estimates for total ANA and anti-DFS70 antibodies are reported for the full population and selected subgroups in **Table 2** and for additional subgroups in **Supplemental Table S4**; the subgroup estimates were essentially averaged over time periods.

**Table 2:** Prevalence estimates for total ANA and anti-DFS70 antibody positivity overall and in selected subgroups.

Factor of lnterest	Subgroup	Prevalence as a Percent (95% CI) of Positivity for: ^a^	
		Total ANA	Anti-DFS70
Overall	All participants	13.0 (12.1, 13.9)	2.76 (2.32, 3.29)
Time Period	1988-1991	11.0 ( 9.7, 12.5)	1.57 (1.20, 2.04)
	1999-2004	11.4 (10.2, 12.8)	2.48 (1.85, 3.33)
	2011-2012	16.1 (14.5, 17.9)	4.04 (3.12, 5.23)
Sex	Males	8.2 ( 7.4, 9.1)	1.42 (0.96, 2.09)
	Females	17.4 (16.2, 18.8)	4.02 (3.37, 4.78)
Age (years)	Adolescents (12-19)	9.2 ( 7.6, 11.0)	2.18 (1.60, 2.95)
	Younger adults (20-49)	10.9 ( 9.8, 12.0)	2.87 (2.37, 3.48)
	Older adults (≥50)	17.6 (16.0, 19.4)	2.84 (2.07, 3.88)
Race/Ethnicity	White persons	12.8 (11.7, 13.9)	2.95 (2.34, 3.71)
	Black persons	15.9 (14.3, 17.5)	1.94 (1.54, 2.44)
	Mexican Americans	12.2 (11.0, 13.5)	2.60 (1.91, 3.53)
	Others	12.0 (10.1, 14.2)	2.48 (1.52, 4.02)
Body Mass Index (BMI)	Underweight/healthy	12.4 (11.4, 13.5)	2.48 (1.96, 3.13)
	Overweight	12.5 (11.2, 14.0)	2.68 (2.06, 3.47)
	Obese	14.2 (12.4, 16.2)	3.23 (2.22, 4.69)
Poverty Income Ratio (PIR)	At/above poverty (≥l)	13.4 (12.4, 14.5)	2.93 (2.38, 3.62)
	Below poverty (<l)	11.7 (10.2, 13.3)	2.45 (1.72, 3.48)
Current Smoking Exposure (ng/ml of cotinine)	None (<0.05)	16.0 (14.2, 18.0)	4.14 (3.19, 5.36)
	Secondhand (0.05-15)	12.5 (11.3, 13.8)	2.77 (2.23, 3.44)
	Active (>15)	9.4 ( 8.2, 10.9)	0.86 (0.58, 1.27)
Alcohol Consumption (age ≥20 years)	None	16.6 (14.6, 18.8)	3.57 (2.74, 4.64)
	Light	13.6 (12.3, 15.1)	2.90 (2.21, 3.80)
	Moderate/heavy	9.5 ( 8.0, 11.3)	2.02 (1.43, 2.83)

ANA, antinuclear antibodies; Anti-DFS70, subclass of ANA that had the DFS staining pattern and bound the 70 kDa DFS protein in an enzyme-linked immunosorbent assay; CI, confidence interval; DFS, dense fine speckled.

^a^ Prevalence was estimated under a logistic regression model for total ANA or anti-DFS70 antibody positivity and expressed as the percent positive. Each model adjusted for the survey design variables (sampling strata, clusters, and weights) and included a categorical covariate for the factor of interest.

**Figure 1:**
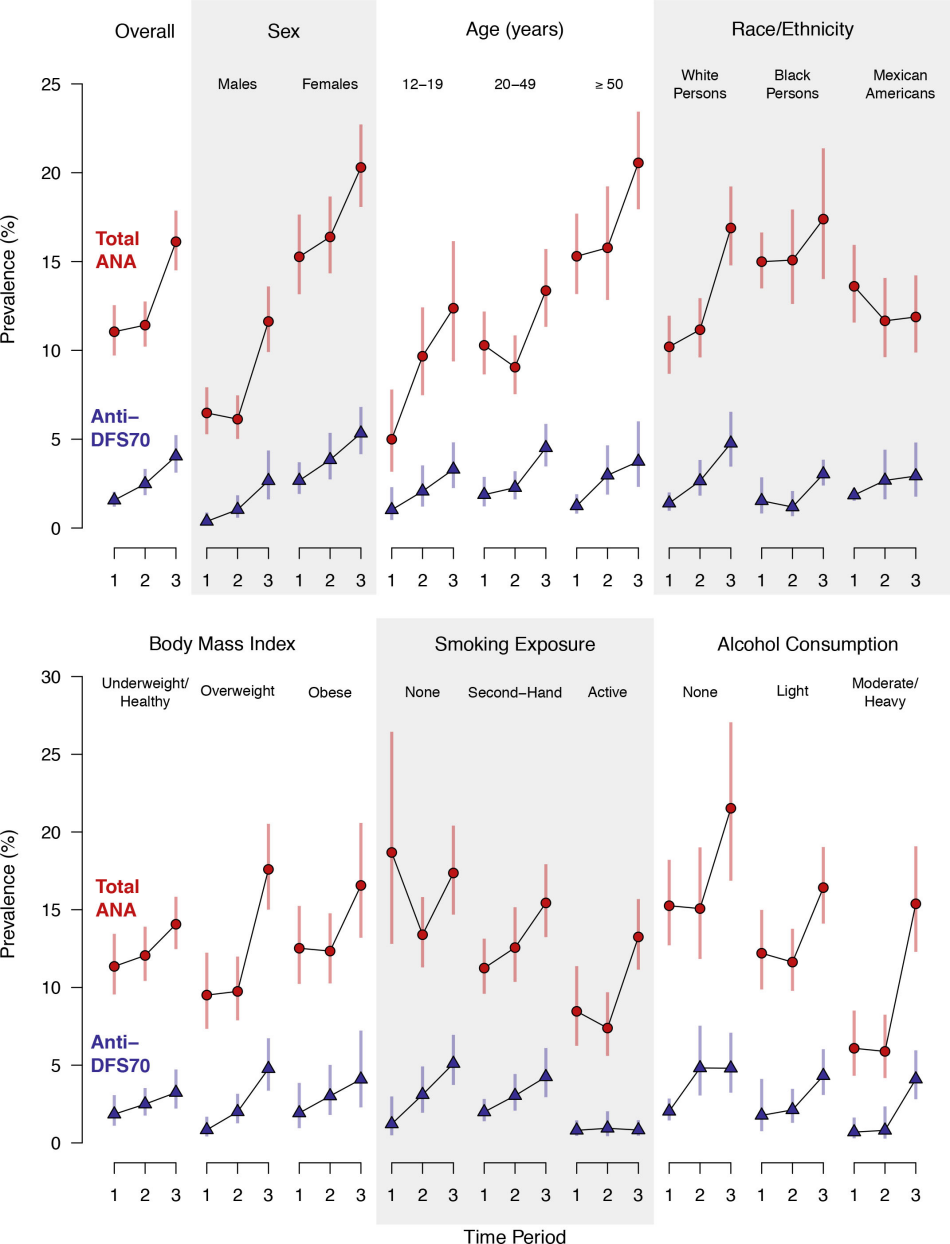
Prevalence of antinuclear antibodies (ANA) and anti-dense fine speckled 70 (DFS70) antibodies in the US population ≥12 years old and in selected subgroups. Separate estimates are plotted for Period 1 (1988–1991), Period 2 (1999–2004), and Period 3 (2011–2012). The prevalence estimates and 95% confidence intervals (CIs) for total ANA are represented by red circles and red vertical lines, respectively, while the anti-DFS70 antibody prevalence estimates and 95% CIs are represented by blue triangles and blue vertical lines. Period-specific prevalence estimates are connected by black lines to visualize time trends. The estimates were derived from a logistic regression model for total ANA or anti-DFS70 antibody positivity, which stratified by the factor defining the subgroup and adjusted for the survey-design variables (sampling strata, clusters, and weights) and a categorical covariate for time period. Participants with missing data for the factor defining the subgroup were excluded only from that subgroup analysis.

In contrast, period-specific prevalence estimates are shown for the full population and selected subgroups in **Figure 1** (and additional subgroups in **Supplemental Figure S1**). The full population estimates in **Figure 1** are visual representations of the numerical values given for time period in **Table 2**, but the subgroup estimates in **Figure 1** and **Supplemental Figure S1** are period-specific, while those in **Table 2** and **Supplemental Table S4** are not. These plots clearly illustrate previously noted increases in total ANA positivity over time (15) and several known total ANA associations, including higher prevalence in females, older adults, and black persons (10, 15). Some, but not all, of these relationships are also seen for anti-DFS70 antibodies. For example, as with total ANA, the estimated prevalence of anti-DFS70 antibodies increased over time and was higher in females, but unlike total ANA, it was not higher in black persons and appeared unrelated to age.

### Correlates

Covariate-adjusted analyses replicated previously observed (10, 15) ANA associations with time period (P<0.0001), sex (P<0.0001), age (P<0.0001), and race/ethnicity (P=0.0008) (**Table 3**), with higher total ANA prevalence in the last period, females, older adults, and black persons. Period and sex were also associated with anti-DFS70 antibodies (P<0.0001), but age was not (P=0.168), and the evidence for race/ethnicity was marginal (P=0.015) and suggested lower rather than higher prevalence in black persons. However, there was strong evidence that current smoking exposure was inversely associated with anti-DFS70 antibody prevalence (P<0.0001), in contrast to the weak evidence for total ANA (P=0.046). No other factor was clearly associated with total ANA or anti-DFS70 antibodies (**Table 3** and **Supplemental Table S5**).

**Table 3:** Covariate-adjusted odds ratios for assessing associations between selected factors and total ANA, anti-DFS70 antibodies, and other ANA.

Factor of Interest	Category	P-value or Odds Ratio (95% CI) of Positivity for: ^a^		
		Total ANA	Anti-DFS70	Other ANA
Time Period	1988-1991	P<0.0001	P<0.0001	P=0.0004
	1999-2004	1.02 (0.84, 1.24)	1.66 (1.11, 2.48)	0.92 (0.76, 1.12)
	2011-2012	1.51 (1.24, 1.84)	2.83 (1.93, 4.13)	1.28 (1.06, 1.55)
Sex	Males	P<0.0001	P<0.0001	P<0.0001
	Females	2.32 (2.03, 2.66)	2.97 (1.97, 4.48)	2.13 (1.83, 2.49)
Age (years)	Adolescents (12-19)	P<0.0001	P=0.168	P<0.0001
	Younger adults (20-49)	1.22 (0.98, 1.50)	1.31 (0.97, 1.77)	1.18 (0.93, 1.51)
	Older adults (≥50)	2.06 (1.64, 2.61)	1.13 (0.74, 1.73)	2.34 (1.81, 3.04)
Race/Ethnicity	White persons	P=0.0008	P=0.015	P<0.0001
	Black persons	1.38 (1.18, 1.60)	0.60 (0.45, 0.82)	1.63 (1.38, 1.93)
	Mexican Americans	1.14 (0.95, 1.36)	0.80 (0.52, 1.21)	1.25 (1.03, 1.52)
	Others	0.96 (0.76, 1.21)	0.70 (0.38, 1.28)	1.05 (0.83, 1.33)
Body Mass Index (BMI)	Underweight/healthy	P=0.868	P=0.441	P=0.544
	Overweight	0.96 (0.82, 1.12)	1.17 (0.77, 1.78)	0.92 (0.77, 1.09)
	Obese	1.00 (0.85, 1.19)	1.26 (0.86, 1.83)	0.95 (0.80, 1.13)
Poverty Income Ratio (PIR)	At/above poverty (≥l)	P=0.056	P=0.395	P=0.068
	Below poverty (<l)	0.83 (0.68, 1.01)	0.83 (0.53, 1.29)	0.84 (0.69, 1.01)
Current Smoking Exposure (ng/ml of cotinine)	None (<0.05)	P=0.046	P<0.0001	P=0.740
	Secondhand (0.05-15)	1.01 (0.85, 1.20)	1.03 (0.75, 1.43)	1.01 (0.85, 1.20)
	Active (>15)	0.75 (0.59, 0.96)	0.28 (0.18, 0.43)	0.92 (0.71, 1.18)
Alcohol Consumption (age ≥20 years)	None	P=0.058	P=0.091	P=0.128
	Light	0.95 (0.77, 1.19)	0.66 (0.43, 1.01)	1.06 (0.83, 1.35)
	Moderate/heavy	0.75 (0.59, 0.96)	0.59 (0.36, 0.96)	0.81 (0.64, 1.03)

ANA, antinuclear antibodies; Anti-DFS70, subclass of ANA that had the DFS staining pattern and bound the 70 kDa DFS protein in an enzyme-linked immunosorbent assay; CI, confidence interval; DFS, dense fine speckled; Other ANA, all types of ANA except anti-DFS70 antibodies.

^a^ Each association was assessed by estimating an odds ratio under a logistic regression model for the prevalence of Total ANA, Anti-DFS70, or Other ANA. The model adjusted for the survey design variables (sampling strata, clusters, and weights), a restricted cubic spline in age, and categorical covariates for time period, sex, race/ethnicity, and the factor of interest. The first category of each factor is the referent; thus, its odds ratio is 1.00 by definition and is not shown. Instead, we show a P-value that indicates the statistical significance of the association between the factor as a whole and antibody status, based on an F-test from a statistical contrast.

Total ANA prevalence increased in the last time period relative to the first (OR=1.51; 95% CI=1.24-1.84), but the positive time trend for anti-DFS70 antibody prevalence was even steeper, with OR=1.66 (95% CI=1.11-2.48) in 1999-2004 and OR=2.83 (95% CI=1.93-4.13) in 2011-2012. Also, while the odds of having ANA in general were over two times higher in females than males (OR=2.32; 95% CI=2.03-2.66), the odds of specifically having anti-DFS70 antibodies were roughly three times higher in females (OR=2.97; 95% CI=1.97-4.48). The prevalence of total ANA increased with age, with OR=1.22 (95% CI=0.98-1.50) for younger adults relative to adolescents and OR=2.06 (95% CI=1.64-2.61) for older adults, but there was little evidence of an age association with anti-DFS70 antibodies. Black persons had a higher prevalence of total ANA than white persons (OR=1.38; 95% CI=1.18-1.60), but a lower prevalence of anti-DFS70 antibodies (OR=0.60; 95% CI=0.45-0.82). Three-fourths as many active smokers had ANA in general relative to nonsmokers (OR=0.75; 95% CI=0.59-0.96), but that proportion was close to one-fourth for anti-DFS70 antibodies specifically (OR=0.28; 95% CI=0.18-0.43). All association results are shown in **Table 3** and **Supplemental Table S5**.

### Time trends

There was strong evidence (P<0.0001) that the overall prevalence of both total ANA and anti-DFS70 antibodies increased over time (**Table 4**). Adjusted for covariates, the estimated ORs for total ANA in the second and third periods relative to the first were 1.02 (95% CI=0.84-1.24) and 1.51 (95% CI=1.24-1.84), respectively, and the corresponding OR estimates for anti-DFS70 antibodies were 1.66 (95% CI=1.11-2.48) and 2.83 (95% CI=1.93-4.13).

**Table 4:** Covariate-adjusted assessments of time trends for total ANA, anti-DFS70 antibodies, and other ANA in selected subgroups^a^.

Factor of lnterest	Subgroup	Response	Prevalence Odds Ratio (95% CI) for Time Period			Trend
			1988-1991	1999-2004	2011-2012	P-value
Overall	All participants	Total ANA	1.00	1.02 (0.84, 1.24)	1.51 (1.24, 1.84)	<0.0001
		Anti-DFS70	1.00	1.66 ( 1.11, 2.48)	2.83 (1.93, 4.13)	<0.0001
		Other ANA	1.00	0.92 (0.76, 1.12)	1.28 (1.06, 1.55)	0.010
Sex	Males	Total ANA	1.00	0.91 (0.68, 1.23)	1.78 (1.34, 2.38)	0.0001
		Anti-DFS70	1.00	2.80 (1.00, 7.89)	7.40 (2.63, 20.8)	0.0003
		Other ANA	1.00	0.80 (0.58, 1.10)	1.43 (1.06, 1.92)	0.016
	Females	Total ANA	1.00	1.07 (0.84, 1.36)	1.37 (1.09, 1.73)	0.008
		Anti-DFS70	1.00	1.51 (0.93, 2.45)	2.20 (1.44, 3.35)	0.0004
		Other ANA	1.00	0.98 (0.77, 1.24)	1.20 (0.94, 1.52)	0.139
Age (years)	Adolescents (12-19)	Total ANA	1.00	2.07 (1.18, 3.64)	2.77 (1.56, 4.91)	0.0004
		Anti-DFS70	1.00	2.09 (0.77, 5.66)	3.43 (1.36, 8.67)	0.006
		Other ANA	1.00	2.05 (1.01, 4.16)	2.53 (1.22, 5.24)	0.008
	Younger adults (20-49)	Total ANA	1.00	0.86 (0.64, 1.14)	1.32 (1.00, 1.74)	0.067
		Anti-DFS70	1.00	1.23 (0.69, 2.18)	2.47 (1.44, 4.24)	0.001
		Other ANA	1.00	0.78 (0.56, 1.09)	1.06 (0.80, 1.39)	0.788
	Older adults (≥50)	Total ANA	1.00	1.06 (0.79, 1.44)	1.50 (1.16, 1.93)	0.002
		Anti-DFS70	1.00	2.56 (1.36, 4.85)	3.37 (1.72, 6.61)	0.003
		Other ANA	1.00	0.93 (0.68, 1.27)	1.32 (1.00, 1.74)	0.038
Race/Ethnicity	White persons	Total ANA	1.00	1.08 (0.85, 1.39)	1.73 (1.35, 2.21)	<0.0001
		Anti-DFS70	1.00	1.95 (1.16, 3.28)	3.65 (2.27, 5.87)	<0.0001
		Other ANA	1.00	0.95 (0.74, 1.21)	1.40 (1.11, 1.76)	0.005
	Black persons	Total ANA	1.00	0.96 (0.74, 1.23)	1.08 (0.81, 1.44)	0.584
		Anti-DFS70	1.00	0.73 (0.30, 1.74)	1.89 (0.96, 3.72)	0.053
		Other ANA	1.00	0.99 (0.75, 1.30)	1.00 (0.73, 1.35)	0.983
	Mexican Americans	Total ANA	1.00	0.81 (0.61, 1.07)	0.83 (0.61, 1.12)	0.268
		Anti-DFS70	1.00	1.46 (0.81, 2.61)	1.64 (0.94, 2.87)	0.162
		Other ANA	1.00	0.72 (0.53, 0.98)	0.71 (0.47, 1.06)	0.123
	Others	Total ANA	1.00	0.71 (0.33, 1.55)	1.12 (0.55, 2.25)	0.504
		Anti-DFS70	1.00	0.76 (0.15, 3.90)	0.63 (0.14, 2.82)	0.527
		Other ANA	1.00	0.70 (0.30, 1.66)	1.27 (0.59, 2.72)	0.290
Body Mass Index (BMI)	Underweight/healthy	Total ANA	1.00	1.04 (0.81, 1.35)	1.25 (0.97, 1.60)	0.097
		Anti-DFS70	1.00	1.37 (0.72, 2.61)	1.88 (0.95, 3.70)	0.069
		Other ANA	1.00	0.98 (0.75, 1.28)	1.12 (0.88, 1.42)	0.405
	Overweight	Total ANA	1.00	1.02 (0.71, 1.45)	2.01 (1.41, 2.85)	<0.0001
		Anti-DFS70	1.00	2.42 (1.04, 5.61)	5.76 (2.56, 13.0)	<0.0001
		Other ANA	1.00	0.88 (0.61, 1.26)	1.59 (1.08, 2.35)	0.018
	Obese	Total ANA	1.00	1.04 (0.75, 1.43)	1.45 (0.98, 2.14)	0.056
		Anti-DFS70	1.00	1.86 (0.75, 4.59)	2.64 (1.03, 6.72)	0.049
		Other ANA	1.00	0. 90 (0.65, 1.25)	1.24 (0.90, 1.70)	0.137
Poverty Income Ratio (PIR)	At/above poverty (≥1)	Total ANA	1.00	1.00 (0.80, 1.24)	1.58 (1.26, 1.97)	<0.0001
		Anti-DFS70	1.00	1.89 (1.19, 3.02)	3.67 (2.35, 5.75)	<0.0001
		Other ANA	1.00	0.87 (0.69, 1.09)	1.27 (1.02, 1.57)	0.026
	Below poverty (<1)	Total ANA	1.00	0.98 (0.65, 1.47)	1.26 (0.85, 1.86)	0.227
		Anti-DFS70	1.00	0.76 (0.27, 2.14)	0.77 (0.31, 1.91)	0.575
		Other ANA	1.00	1.08 (0.70, 1.66)	1.44 (0.94, 2.20)	0.082
Current Smoking Exposure (ng/ml of cotinine)	None (<0.05)	Total ANA	1.00	0.73 (0.45, 1.18)	1.05 (0.64, 1.71)	0.161
		Anti-DFS70	1.00	2.71 (0.98, 7.49)	5.00 (1.93, 13.0)	0.004
		Other ANA	1.00	0.60 (0.37, 0.98)	0.78 (0.47, 1.28)	0.856
	Secondhand (0.05-15)	Total ANA	1.00	1.23 (0.93, 1.64)	1.66 (1.28, 2.15)	0.0005
		Anti-DFS70	1.00	1.83 (1.10, 3.05)	2.67 (1.56, 4.56)	0.0003
		Other ANA	1.00	1.11 (0.82, 1.49)	1.42 (1.08, 1.88)	0.030
	Active (>15)	Total ANA	1.00	0.82 (0.53, 1.25)	1.47 (1.02, 2.13)	0.061
		Anti-DFS70	1.00	1.13 (0.43, 2.97)	1.20 (0.46, 3.14)	0.703
		Other ANA	1.00	0.78 (0.50, 1.24)	1.48 (0.96, 2.28)	0.103
Alcohol Consumption (age ≥20 years)	None	Total ANA	1.00	0.95 (0.66, 1.37)	1.39 (0.95, 2.02)	0.147
		Anti-DFS70	1.00	2.60 (1.44, 4.67)	2.72 (1.63, 4.55)	<0.0001
		Other ANA	1.00	0.73 (0.51, 1.05)	1.18 (0.81, 1.70)	0.706
	Light	Total ANA	1.00	0.86 (0.62, 1.20)	1.30 (0.95, 1.78)	0.064
		Anti-DFS70	1.00	1.27 (0.46, 3.50)	2.93 (1.16, 7.41)	0.014
		Other ANA	1.00	0.79 (0.57, 1.10)	1.05 (0.74, 1.48)	0.630
	Moderate/heavy	Total ANA	1.00	0.97 (0.58, 1.61)	2.49 (1.58, 3.93)	0.0001
		Anti-DFS70	1.00	1.30 (0.32, 5.25)	6.49 (2.53, 16.7)	0.0002
		Other ANA	1.00	0.92 (0.55, 1.53)	1.97 (1.25, 3.10)	0.003

ANA, antinuclear antibodies; Anti-DFS70, subclass of ANA that had the DFS staining pattern and bound the 70 kDa DFS protein in an enzyme-linked immunosorbent assay; CI, confidence interval; DFS, dense fine speckled; Other ANA, all types of ANA except anti-DFS70 antibodies.

^a^ Time trend assessments were based on two logistic regression models for antibody positivity, which adjusted for the survey design variables (sampling strata, clusters, and weights), a restricted cubic spline in age, and categorical covariates for sex and race/ethnicity. The first model added a categorical covariate for time period and estimated the prevalence odds ratio for each period, relative to the earliest period. The second model added a quantitative covariate for the number of years between period midpoints, relative to the earliest period, and produced a P-value from a χ2-test to assess a time trend.

In subgroup analyses, the increasing time trend for total ANA was seen in both males (P=0.0001) and females (P=0.008), as well as in adolescents (P=0.0004) and adults ≥50 years old (P=0.002), but not adults 20-49 years old (**Table 4**). The positive time trend for total ANA was also observed in white persons (P<0.0001), overweight individuals (P<0.0001), persons living at or above the poverty level (P<0.0001), persons exposed to secondhand tobacco smoke (P=0.0005), and moderate to heavy drinkers (P=0.0001) (**Table 4**), as well as in persons for whom poor health did (P=0.004) or did not (P=0.001) prevent them from working (**Supplemental Table S6**). These time trends for total ANA are depicted in **Figure 1** and **Supplemental Figure S1**.

Increases in anti-DFS70 antibodies over time were seen in most of the same subgroups: males (P=0.0003), females (P=0.0004), adolescents (P=0.006), older adults (P=0.003), white persons (P<0.0001), overweight individuals (P<0.0001), individuals living at or above the poverty level (P<0.0001), persons exposed to secondhand tobacco smoke (P=0.0003), and moderate to heavy drinkers (P=0.0002). Unlike total ANA, though, the positive anti-DFS70 antibody time trend was also seen in younger adults (P=0.001), nonsmokers (P=0.004), nondrinkers (P<0.0001), and persons with very high C-reactive protein levels (P<0.0001) (**Table 4**; **Supplemental Table S6**; **Figure 1**; **Supplemental Figure S1**).

We assessed whether the time trends in subgroups defined by a given factor’s categories were different or statistically consistent with the overall time trend. We found marginal evidence (0.001<P<0.05) that the ANA time trends for subgroups defined by age (P=0.036), race/ethnicity (P=0.002), and alcohol consumption (P=0.014) differed, as did the anti-DFS70 antibody time trends for subgroups defined by sex (P=0.025), PIR (P=0.005), current smoking exposure (P=0.034), and C-reactive protein (P=0.002) (**Supplemental Table S7**).

### Additional analyses

We investigated whether anti-DFS70 antibody correlates and time trends systematically differed from those for total ANA by analyzing “other” ANA (i.e., any type of ANA except anti-DFS70 antibodies). There was weak evidence that current smoking exposure was inversely associated with total ANA (P=0.046), strong evidence for anti-DFS70 antibodies (P<0.0001), and no evidence for other ANA (P=0.740), suggesting that anti-DFS70 antibodies were driving the inverse association between total ANA and smoking (**Table 3**). As for increases in prevalence across periods, the overall positive time trend for other ANA was smaller than that for total ANA and anti-DFS70 antibodies, suggesting that the anti-DFS70 antibody time trend contributed to the trend for total ANA (**Table 4**). Similar evidence of this relationship was seen in both sexes, older adults, white persons, overweight individuals, individuals at or above the poverty level, persons exposed to secondhand tobacco smoke, and moderate to heavy drinkers (**Table 4**), as well as in persons with more than a high school education (**Supplemental Table S6**).

Smoking exposure decreased over the 25-year span of our study and was inversely associated with anti-DFS70 antibodies, which could confound the observed anti-DFS70 antibody time trends. Thus, we repeated our covariate-adjusted analyses after adding a covariate for smoking exposure and found that, relative to Period 1, anti-DFS70 antibody prevalence increased in Period 2 (OR=1.65; 95% CI=1.08-2.52) and again in Period 3 (OR=2.69; 95% CI=1.80-4.02). These odds ratios are similar to those from our original analysis, which adjusted for sex, age, and race/ethnicity, but not smoking exposure (**Table 3**). In both analyses, the statistical evidence of a positive time trend was strong (P<0.0001), suggesting that changes in smoking over this 25-year span cannot explain the increase in anti-DFS70 antibody prevalence over time.

## Discussion

Our main goal was to estimate the prevalence of anti-DFS70 antibodies, identify factors associated with them, and assess changes over time. A secondary goal was to evaluate whether subgroup time trends were heterogeneous, as a way of discovering possible drivers or protective factors that could steepen or flatten the general trend. A tertiary goal was to compare our results for anti-DFS70 antibodies with established results for total ANA. In view of these goals, we summarize our findings below, after a brief review of the relevance of anti-DFS70 antibodies. We also discuss strengths and limitations of our study and several other points of interest.

The standard HEp-2 IFA for ANA detects a heterogeneous group of autoantibodies and is a common screening tool when a clinical presentation suggests a possible autoimmune disease (17). However, relatively little is known about the natural history of ANA in the absence of an autoimmune disease. Staining patterns are important for understanding the relevance of ANA in pre-clinical, symptomatic, and healthy populations. Recently, thirty ANA patterns identified by alphanumeric codes (AC-0 to AC-29) were described and classified by the committee for the International Consensus on ANA Patterns (ICAP) (18, 30), and the clinical relevance was summarized online (www.ANApatterns.org) (17). The DFS (AC-2) staining pattern is associated with anti-DFS70 antibodies, which are more common in healthy individuals than in those with SARD (16, 31). The autoantigen DFS70 is a transcription co-activator regulating the expression of oxidative-stress, inflammatory, and antioxidant genes (32). The consensus is that individuals with high-titer monospecific anti-DFS70 antibodies (i.e., no other well-known autoantibodies that are markers for autoimmune diseases) seldom have SARD (16, 17, 24–27).

The selection of the anti-DFS70 ELISA used in this study was based on its commercial availability at the time and the overall laboratory experience with alternatives for anti-DFS70 testing (33). A natural question is why some samples were positive in the DFS/AC-2 IFA but negative in the anti-DFS70 ELISA. Further study focused primarily on the few higher-titered DFS/AC-2 positive samples. We attempted to identify the target antigen(s) in these sera using Western blot and immunoprecipitation-mass spectrometry, as described in the characterization of other autoantibody reference materials (33–35). No target antigen was consistently identified, though the data clearly showed that these sera did not recognize DFS70 in either Western blot or immunoprecipitation-mass spectrometry. These sera may be similar to some that the community refers to as showing a “pseudo DFS” pattern. Further work is needed to characterize these sera as “pseudo DFS” in the absence of a well-defined autoantigen target or clinical relevance. Our observations are consistent with a recent publication that reports sera with a DFS-like or pseudo-DFS pattern did not react with DFS70, which suggests they may recognize diverse proteins or conformational epitopes (19). The percentage of sera negative for anti-DFS70 in the DFS/AC-2 pool is expected to vary substantially in different laboratories, based on many factors. Harmonization will need to consider variations in experience reading IFA, selection of a validated anti-DFS70 assay, and proper use of available anti-DFS70 reference material (33).

The large NHANES databases and serum repositories allowed us to explore anti-DSF70 antibody prevalence, correlates, and time trends in nationally representative samples of the civilian noninstitutionalized US population ≥12 years old. Anti-DFS70 antibody prevalence increased from 1.6% in 1988-1991 to 2.5% in 1999-2004 to 4.0% in 2011-2012, which correspond to 3.2 million, 5.8 million, and 10.4 million seropositive individuals, respectively. Thus, over this 25-year span, we observed a 2.5-fold increase in the prevalence for anti-DFS70 antibodies (1.6% to 4.0%) compared with a 1.5-fold increase for total ANA (11.0% to 16.1%). Analyses accounting for sex, age, and race/ethnicity showed this positive trend was statistically significant (P<0.0001), with anti-DFS70 antibody prevalence being 66% higher in 1999-2004 (1.66-fold increase) and 183% higher in 2011-2012 (2.83-fold increase), relative to 1988-1991. Covariate-adjusted analyses also revealed anti-DFS70 antibody associations with sex and smoking (both P<0.0001), where the odds of having anti-DFS70 antibodies were three times higher in females than in males and one-fourth as high in active smokers as in nonsmokers. There was also some evidence of a race/ethnicity association (P=0.015), with black persons only 60% as likely as white persons to have anti-DFS70 antibodies. We did not find clear evidence of anti-DFS70 antibody associations with other factors considered.

Both total ANA and anti-DFS70 antibodies increased significantly over time and both were associated with sex and race/ethnicity, but the clear age association with total ANA was not apparent for anti-DFS70 antibodies, and the obvious smoking association with anti-DFS70 antibodies was only weakly supported for total ANA. Our analysis of other ANA suggested anti-DFS70 antibodies may have driven the inverse association between total ANA and smoking, but played little to no role in the more obvious ANA associations with sex, age, and race/ethnicity.

We also investigated whether the overall anti-DFS70 antibody time trend was seen in subgroups. Anti-DFS70 antibody prevalence increased over time in both sexes, all three age groups, white persons, overweight individuals, persons at or above the poverty level, nonsmokers, persons exposed to secondhand smoke, alcohol abstainers, moderate to heavy alcohol drinkers, persons with more than a high school education, and persons with very high C-reactive protein levels. In many of these subgroups, similar time trends were observed for the broader category of total ANA. However, there was little evidence of a time trend for total ANA in younger adults, nonsmokers, alcohol abstainers, or persons with very high C-reactive protein levels. It is not clear why some association patterns for anti-DSF70 antibodies differ from those for total ANA, but distinct mechanisms could be at play, as has been hypothesized to explain why smoking is a risk factor for some diseases but protective for others (36).

Our study had several strengths. Total ANA and anti-DFS70 antibody cohorts were large, spanned 25 years, and were weighted to be representative of the civilian noninstitutionalized US population ≥12 years old. All assays were performed in the same laboratory and used the same evaluators, methods, and equipment. Our analyses accounted for many sociodemographic factors and health conditions as potential correlates or modifiers. We focused on persons confirmed positive for anti-DFS70 antibodies by ELISA among those screened positive for DFS staining by IFA; this is consistent with the standard protocol for a typical clinical immunology laboratory, which usually only screens for anti-DFS70 antibodies in sera with positive DFS staining.

Our findings are also subject to limitations: 1) the NHANES excluded institutionalized persons, such as the elderly in residential care; 2) statistical analyses were based on cross-sectional data rather than longitudinal data with repeated measures; 3) neither total ANA nor anti-DFS70 antibodies were assessed in children <12 years old; 4) some information was self-reported; 5) not all participants were tested for anti-DFS70 antibodies (i.e., only those positive for ANA with the DFS staining pattern); and 6) there were inadequate clinical data available to allow for disease association studies.

Some individuals may have had anti-DFS70 antibodies but did not test positive for DFS (AC-2) staining if there were other autoantibody signals that hindered the detection of the DFS pattern. For example, if someone had both anti-DFS70 and anti-DNA antibodies, the anti-DNA antibodies could have produced a strong homogeneous staining pattern that obscured the DFS pattern; however, there were few participants with strong homogeneous staining in the NHANES cohorts. We did not perform the ELISA test for anti-DFS70 antibodies among non-DFS-positive participants due to cost considerations and because most clinical laboratories do not test for anti-DFS70 antibodies without prior HEp-2 IFA pre-screening. However, when a clear DFS pattern was observed, it was likely from high-titer anti-DFS70 antibodies (37). Thus, our findings relate mostly to high-titer or monospecific anti-DFS70 antibodies, which have been reported mainly in non-SARD or healthy individuals (38), and therefore our analyses probably underestimated the true prevalence of anti-DFS70 antibodies.

As discussed above and reviewed by others (32, 39), the search for clinical associations between anti-DFS70 antibodies and well-defined disease conditions has not yielded clear results. There is general agreement that high-titer monospecific anti-DFS70 antibodies tend to be associated with non-SARD conditions, including certain gynecologic syndromes, atopic disorders, skin disorders (psoriasis and alopecia areata), fibromyalgia/chronic pain syndrome/chronic fatigue syndrome, Hashimoto’s thyroiditis, and prostate cancer (38, 40–42). However, recent studies have also shown anti-DFS70 antibodies in persons with SARD, including systemic lupus erythematosus, rheumatoid arthritis, undifferentiated connective tissue disease, and Sjögren’s Syndrome (38, 40). Our data revealed a substantial prevalence of anti-DFS70 antibodies in the NHANES cohorts, and increasingly so in the more recent cohorts. Perhaps anti-DFS70 antibodies are not as strongly linked to a particular disease as they are to specific, but yet to be fully defined, exposures in our rapidly changing environment (8). One possible explanation for the weak association between anti-DFS70 antibodies and particular diseases may be the increasingly high relative prevalence of anti-DFS70 antibodies in the general population, as shown in the present study. In a future study, we plan to investigate possible associations between anti-DFS70 antibodies and additional environmental exposures and health conditions.

In conclusion, anti-DFS70 antibodies in the US were associated with sex and exposure to tobacco smoke, as well as the period of evaluation. Specifically, their prevalence was higher in females, lower in active smokers, and increased substantially over the 25-year span of our study. This positive time trend was observed in both the general population and many subgroups. We also found some evidence of an association between race/ethnicity and anti-DFS70 antibodies, with black persons having a lower prevalence than white persons. The known age association with total ANA, however, was not obvious for anti-DFS70 antibodies. More research is needed to understand the risk factors, pathologic or potentially protective influences on disease (43), and clinical implications of anti-DFS70 antibodies.

## Data availability statement

All of the NHANES data that we analyzed in this study are freely and publicly available from the CDC web sites.

## Ethics statement

The NHANES protocol was approved by the Human Subjects Institutional Review Board of the US Centers for Disease Control and Prevention (CDC). The patients/participants provided their written informed consent to participate in this study.

## Author contributions

All authors were involved in drafting the paper or revising it critically for important intellectual content. EC had full access to the data and takes responsibility for its integrity and the accuracy of the data analysis. Study conception and design: GD, BZ, CP, CW, FM, and EC. Acquisition of data: GD, BZ, CC, FM, and EC. Analysis and interpretation of data: GD, BZ, CC, CP, CW, FM, and EC. All authors contributed to the article and approved the submitted version.

## Supplementary material

Supplementary Figure 1 Prevalence of antinuclear antibodies (ANA) and anti-dense fine speckled 70 (DFS70) antibodies in additional subgroups. Separate estimates are plotted for Period 1 (1988–1991), Period 2 (1999–2004), and Period 3 (2011–2012). The prevalence estimates and 95% confidence intervals (CIs) for total ANA are represented by red circles and red vertical lines, respectively, while the anti-DFS70 antibody prevalence estimates and 95% CIs are represented by blue triangles and blue vertical lines. Period-specific prevalence estimates are connected by black lines to visualize time trends. The estimates were derived from a logistic regression model for total ANA or anti-DFS70 antibody positivity, which stratified by the factor defining the subgroup and adjusted for the survey-design variables (sampling strata, clusters, and weights) and a categorical covariate for time period. Participants with missing data for the factor defining the subgroup were excluded only from that subgroup analysis.
