# Supplementary figures and images for: Anti-dense fine speckled 70 (DFS70) autoantibodies: correlates and increasing prevalence in the United States
Source: Front Immunol. 2023 Jun 23;14:1186439. doi: 10.3389/fimmu.2023.1186439 (PMC10326272; doi:10.3389/fimmu.2023.1186439)

Supplemental Figure S1

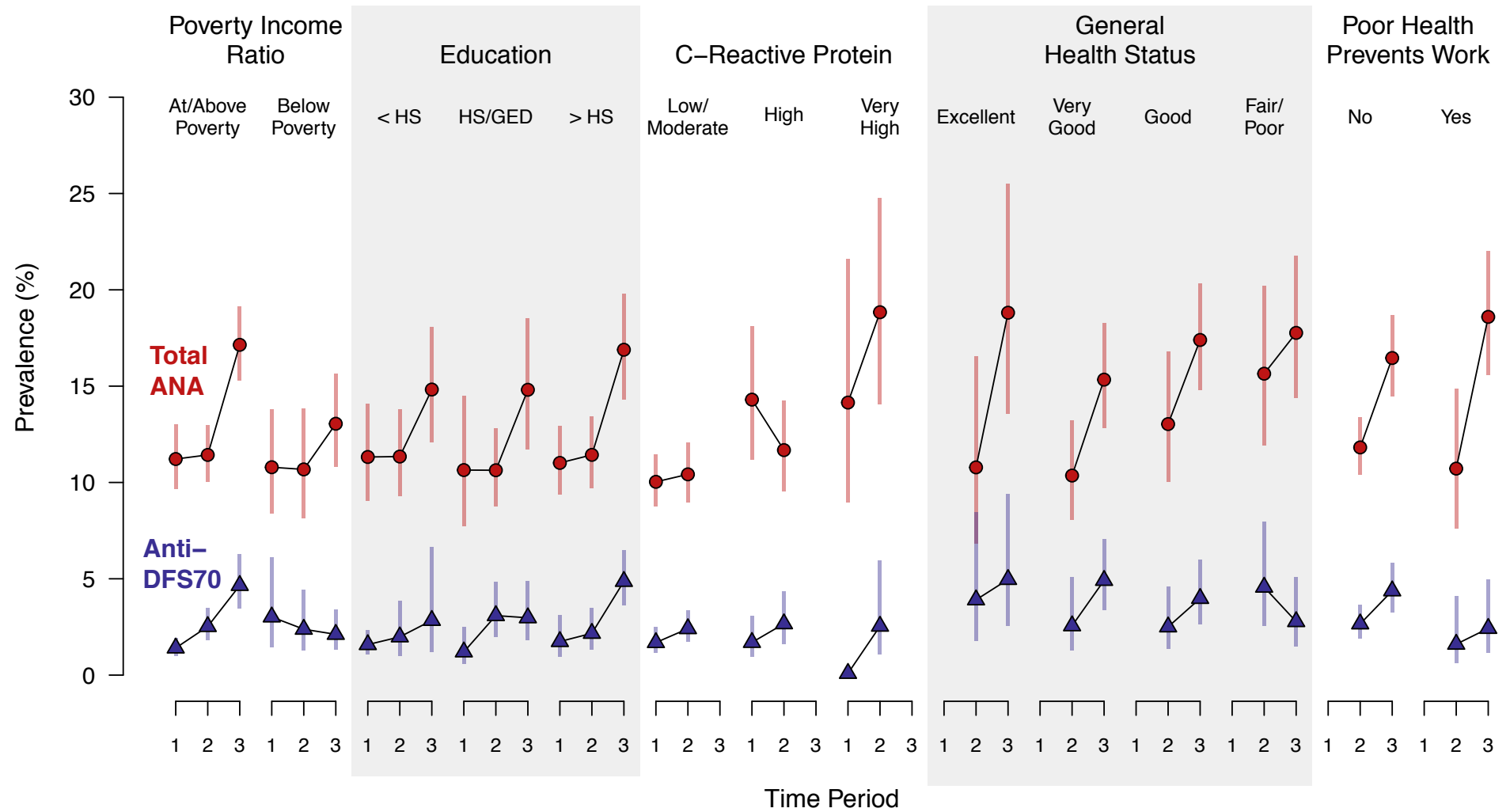

Supplement: Supplementary Figure 1 — Prevalence of antinuclear antibodies (ANA) and anti-dense fine speckled 70 (DFS70) antibodies in additional subgroups. Separate estimates are plotted for Period 1 (1988–1991), Period 2 (1999–2004), and Period 3 (2011–2012). The prevalence estimates and 95% confidence intervals (CIs) for total ANA are represented by red circles and red vertical lines, respectively, while the anti-DFS70 antibody prevalence estimates and 95% CIs are represented by blue triangles and blue vertical lines. Period-specific prevalence estimates are connected by black lines to visualize time trends. The estimates were derived from a logistic regression model for total ANA or anti-DFS70 antibody positivity, which stratified by the factor defining the subgroup and adjusted for the survey-design variables (sampling strata, clusters, and weights) and a categorical covariate for time period. Participants with missing data for the factor defining the subgroup were excluded only from that subgroup analysis. [file DataSheet_1.pdf]
