# Supplementary material for: Anti-dense fine speckled 70 (DFS70) autoantibodies: correlates and increasing prevalence in the United States
Source: Front Immunol. 2023 Jun 23;14:1186439. doi: 10.3389/fimmu.2023.1186439 (PMC10326272; doi:10.3389/fimmu.2023.1186439)
Supplement: Supplementary file 2 [file Table_1.docx]

| Supplemental Table S1. Participant characteristics by time period. | | | | | | | | | | |
| --- | --- | --- | --- | --- | --- | --- | --- | --- | --- | --- |
|  |  |  |  |  |  |  |  |  |  |  |
|  |  |  |  |  |  |  |  |  |  |  |
|  |  |  |  |  |  |  |  |  |  |  |
|  |  |  |  | Number (%) of Participants in Factor Category ^a^ | | | | | | |
|  |  |  |  | Period 1: |  | Period 2: |  | Period 3: |  | All Periods |
| Factor of Interest |  | Category |  | 1988-1991 |  | 1999-2004 |  | 2011-2012 |  | Combined |
|  |  |  |  |  |  |  |  |  |  |  |
| Overall |  | All participants |  | 4,727 (100) |  | 4,527 (100) |  | 4,265 (100) |  | 13,519 (100) |
|  |  |  |  |  |  |  |  |  |  |  |
| Sex |  | Males |  | 2,363 (50) |  | 2,180 (48) |  | 2,098 (49) |  | 6,641 (49) |
|  |  | Females |  | 2,364 (50) |  | 2,347 (52) |  | 2,167 (51) |  | 6,878 (51) |
|  |  |  |  |  |  |  |  |  |  |  |
| Age (years) |  | Adolescents (12-19) |  | 676 (14) |  | 1,098 (24) |  | 767 (18) |  | 2,541 (19) |
|  |  | Younger adults (20-49) |  | 2,218 (47) |  | 1,827 (40) |  | 1,808 (42) |  | 5,853 (43) |
|  |  | Older adults ( ≥50 ) |  | 1,833 (39) |  | 1,602 (35) |  | 1,690 (40) |  | 5,125 (38) |
|  |  |  |  |  |  |  |  |  |  |  |
| Race/Ethnicity |  | White persons |  | 2,060 (44) |  | 2,060 (46) |  | 1,566 (37) |  | 5,686 (42) |
|  |  | Black persons |  | 1,164 (25) |  | 926 (20) |  | 1,033 (24) |  | 3,123 (23) |
|  |  | Mexican Americans |  | 1,354 (29) |  | 1,158 (26) |  | 504 (12) |  | 3,016 (22) |
|  |  | Others |  | 149 ( 3) |  | 383 ( 8) |  | 1,162 (27) |  | 1,694 (13) |
|  |  |  |  |  |  |  |  |  |  |  |
| Body Mass Index (BMI) |  | Underweight/healthy |  | 2,191 (47) |  | 1,778 (39) |  | 1,555 (37) |  | 5,524 (41) |
|  |  | Overweight |  | 1,483 (31) |  | 1,405 (31) |  | 1,214 (29) |  | 4,102 (31) |
|  |  | Obese |  | 1,036 (22) |  | 1,334 (30) |  | 1,427 (34) |  | 3,797 (28) |
|  |  |  |  |  |  |  |  |  |  |  |
| Poverty Income Ratio (PIR) |  | At/above poverty (≥1) |  | 3,225 (76) |  | 3,239 (77) |  | 2,941 (74) |  | 9,405 (76) |
|  |  | Below poverty (<1) |  | 991 (24) | 981 (fdfdfdfddfdf | 943 (23) |  | 1,009 (26) |  | 2,943 (24) |
|  |  |  |  |  |  |  |  |  |  |  |
| Current Smoking Exposure |  | None (<0.05) |  | 411 ( 9) |  | 1,847 (41) |  | 2,366 (55) |  | 4,624 (35) |
| (ng/ml of cotinine) |  | Secondhand (0.05-15) |  | 2,788 (62) |  | 1,654 (37) |  | 1,039 (24) |  | 5,481 (41) |
|  |  | Active (>15) |  | 1,326 (29) |  | 998 (22) |  | 859 (20) |  | 3,183 (24) |
|  |  |  |  |  |  |  |  |  |  |  |
| Alcohol Consumption |  | None |  | 2,009 (53) |  | 1,011 (35) |  | 814 (29) |  | 3,834 (41) |
| (age ≥20 years) |  | Light |  | 834 (22) |  | 1,184 (41) |  | 1,197 (43) |  | 3,215 (34) |
|  |  | Moderate/heavy |  | 948 (25) |  | 676 (24) |  | 754 (27) |  | 2,378 (25) |
|  |  |  |  |  |  |  |  |  |  |  |
| Education |  | Less than high school |  | 2,115 (45) |  | 1,470 (34) |  | 983 (24) |  | 4,568 (35) |
|  |  | High school/GED |  | 1,261 (27) |  | 1,009 (23) |  | 849 (21) |  | 3,119 (24) |
|  |  | More than high school |  | 1,305 (28) |  | 1,887 (43) |  | 2,253 (55) |  | 5,445 (41) |
|  |  |  |  |  |  |  |  |  |  |  |
| C-reactive Protein (mg/L) |  | Low/moderate (≤3) |  | 3,364 (73) |  | 2,978 (66) |  | ----- |  | 6,342 (69) |
|  |  | High (>3 to 10) |  | 932 (20) |  | 1,124 (25) |  | ----- |  | 2,056 (22) |
|  |  | Very high (>10) |  | 333 ( 7) |  | 425 ( 9) |  | ----- |  | 758 ( 8) |
|  |  |  |  |  |  |  |  |  |  |  |
| General Health Status |  | Excellent |  | ----- |  | 288 (13) |  | 325 (10) |  | 613 (11) |
| (age ≥20 years) |  | Very good |  | ----- |  | 630 (28) |  | 843 (27) |  | 1,473 (27) |
|  |  | Good |  | ----- |  | 801 (36) |  | 1,295 (41) |  | 2,096 (39) |
|  |  | Fair/poor |  | ----- |  | 505 (23) |  | 682 (22) |  | 1,187 (22) |
|  |  |  |  |  |  |  |  |  |  |  |
| Poor Health Prevents Work |  | No |  | ----- |  | 3,008 (88) |  | 2,998 (86) |  | 6,006 (87) |
| (age ≥20 years) |  | Yes |  | ----- |  | 420 (12) |  | 500 (14) |  | 920 (13) |
|  |  |  |  |  |  |  |  |  |  |  |
| Positive for Allergens ^b^ |  | No |  | 751 (44) |  | ----- |  | ----- |  | 751 (44) |
| (age <60 years) |  | Yes |  | 951 (56) |  | ----- |  | ----- |  | 951 (56) |
|  |  |  |  |  |  |  |  |  |  |  |
|  |  |  |  |  |  |  |  |  |  |  |
| Abbreviations: NHANES = National Health and Nutrition Examination Survey. | | | | | | | | | | |
| ^a^ For certain factors, some participants in specific time periods and/or age groups were not surveyed by NHANES, some participants were excluded at the analysis stage so that all time periods would involve the same age groups, and some participants were missing data due to nonresponse or other reasons beyond our control. Details and exact counts are given in Supplemental Table S2. A complete absence of participants in a time period is indicated by dashes (-----). | | | | | | | | | | |
| ^b^ Allergens included Alternaria alternata, Bermuda grass, cat, German cockroach, dust mite, peanut, short ragweed, Russian thistle, rye grass, and white oak. | | | | | | | | | | |

| Supplemental Table S2. Number and percentage of participants who were analyzed, were not surveyed, were excluded, and were missing data by factor of interest and time period. | | | | | | | | | | |
| --- | --- | --- | --- | --- | --- | --- | --- | --- | --- | --- |
|  |  |  |  |  |  |  |  |  |  |  |
|  |  |  |  |  |  |  |  |  |  |  |
|  |  |  |  | Number (%) of Participants Experiencing Event ^a^ | | | | | | |
|  |  |  |  | Period 1: |  | Period 2: |  | Period 3: |  | All Periods |
| Factor of Interest |  | Event |  | 1988-1991 |  | 1999-2004 |  | 2011-2012 |  | Combined |
|  |  |  |  |  |  |  |  |  |  |  |
| Sex, Age, and Race/Ethnicity |  | Analyzed |  | 4,727 (100) |  | 4,527 (100) |  | 4,265 (100) |  | 13,519 (100) |
|  |  | Not surveyed |  | 0 ( 0) |  | 0 ( 0) |  | 0 ( 0) |  | 0 ( 0) |
|  |  | Excluded |  | 0 ( 0) |  | 0 ( 0) |  | 0 ( 0) |  | 0 ( 0) |
|  |  | Missing data |  | 0 ( 0) |  | 0 ( 0) |  | 0 ( 0) |  | 0 ( 0) |
|  |  |  |  |  |  |  |  |  |  |  |
| Body Mass Index (BMI) |  | Analyzed |  | 4,710 (>99) |  | 4,517 (>99) |  | 4,196 ( 98) |  | 13,423 ( 99) |
|  |  | Not surveyed |  | 0 ( 0) |  | 0 ( 0) |  | 0 ( 0) |  | 0 ( 0) |
|  |  | Excluded |  | 0 ( 0) |  | 0 ( 0) |  | 0 ( 0) |  | 0 ( 0) |
|  |  | Missing data |  | 17 ( <1) |  | 10 ( <1) |  | 69 ( 2) |  | 96 ( 1) |
|  |  |  |  |  |  |  |  |  |  |  |
| Poverty Income Ratio (PIR) |  | Analyzed |  | 4,216 ( 89) |  | 4,182 ( 92) |  | 3,950 ( 93) |  | 12,348 ( 91) |
|  |  | Not surveyed |  | 0 ( 0) |  | 0 ( 0) |  | 0 ( 0) |  | 0 ( 0) |
|  |  | Excluded |  | 0 ( 0) |  | 0 ( 0) |  | 0 ( 0) |  | 0 ( 0) |
|  |  | Missing data |  | 511 ( 11) |  | 345 ( 8) |  | 315 ( 7) |  | 1,171 ( 9) |
|  |  |  |  |  |  |  |  |  |  |  |
| Current Smoking Exposure |  | Analyzed |  | 4,525 ( 96) |  | 4,499 ( 99) |  | 4,264 (>99) |  | 13,288 ( 98) |
| (ng/ml of cotinine) |  | Not surveyed |  | 0 ( 0) |  | 0 ( 0) |  | 0 ( 0) |  | 0 ( 0) |
|  |  | Excluded |  | 0 ( 0) |  | 0 ( 0) |  | 0 ( 0) |  | 0 ( 0) |
|  |  | Missing data |  | 202 ( 4) |  | 28 ( 1) |  | 1 ( <1) |  | 231 ( 2) |
|  |  |  |  |  |  |  |  |  |  |  |
| Alcohol Consumption ^b^ |  | Analyzed |  | 3,791 ( 80) |  | 2,871 ( 63) |  | 2,765 ( 65) |  | 9,427 ( 70) |
| (age ≥20 years) |  | Not surveyed |  | 676 ( 14) |  | 1,098 ( 24) |  | 767 ( 18) |  | 2,541 ( 19) |
|  |  | Excluded |  | 0 ( 0) |  | 0 ( 0) |  | 0 ( 0) |  | 0 ( 0) |
|  |  | Missing data |  | 260 ( 6) |  | 558 ( 12) |  | 733 ( 17) |  | 1,551 ( 11) |
|  |  |  |  |  |  |  |  |  |  |  |
| Education |  | Analyzed |  | 4,681 ( 99) |  | 4,366 ( 96) |  | 4,085 ( 96) |  | 13,132 ( 97) |
|  |  | Not surveyed |  | 0 ( 0) |  | 0 ( 0) |  | 0 ( 0) |  | 0 ( 0) |
|  |  | Excluded |  | 0 ( 0) |  | 0 ( 0) |  | 0 ( 0) |  | 0 ( 0) |
|  |  | Missing data |  | 46 ( 1) |  | 161 ( 4) |  | 180 ( 4) |  | 387 ( 3) |
|  |  |  |  |  |  |  |  |  |  |  |
| C-reactive Protein (mg/L) ^c^ |  | Analyzed |  | 4,629 ( 98) |  | 4,527 (100) |  | 0 ( 0) |  | 9,156 ( 68) |
|  |  | Not surveyed |  | 0 ( 0) |  | 0 ( 0) |  | 4,265 (100) |  | 4,265 ( 32) |
|  |  | Excluded |  | 0 ( 0) |  | 0 ( 0) |  | 0 ( 0) |  | 0 ( 0) |
|  |  | Missing data |  | 98 ( 2) |  | 0 ( 0) |  | 0 ( 0) |  | 98 ( 1) |
|  |  |  |  |  |  |  |  |  |  |  |
| General Health Status ^d^ |  | Analyzed |  | 0 ( 0) |  | 2,224 ( 49) |  | 3,145 ( 74) |  | 5,369 ( 40) |
| (age ≥20 years) |  | Not surveyed |  | 4,727 (100) |  | 1,578 ( 35) |  | 0 ( 0) |  | 6,305 ( 47) |
|  |  | Excluded |  | 0 ( 0) |  | 554 ( 12) |  | 767 ( 18) |  | 1,321 ( 10) |
|  |  | Missing data |  | 0 ( 0) |  | 171 ( 4) |  | 353 ( 8) |  | 524 ( 4) |
|  |  |  |  |  |  |  |  |  |  |  |
| Poor Health Prevents Work ^e^ |  | Analyzed |  | 0 ( 0) |  | 3,428 ( 76) |  | 3,498 ( 82) |  | 6,926 ( 51) |
| (age ≥20 years) |  | Not surveyed |  | 4,727 (100) |  | 1,098 ( 24) |  | 767 ( 18) |  | 6,592 ( 49) |
|  |  | Excluded |  | 0 ( 0) |  | 0 ( 0) |  | 0 ( 0) |  | 0 ( 0) |
|  |  | Missing data |  | 0 ( 0) |  | 1 ( <1) |  | 0 ( 0) |  | 1 ( <1) |
|  |  |  |  |  |  |  |  |  |  |  |
| Positive for Allergens ^f^ |  | Analyzed |  | 1,702 ( 36) |  | 0 ( 0) |  | 0 ( 0) |  | 1,702 ( 13) |
| (age <60 years) |  | Not surveyed |  | 1,383 ( 29) |  | 4,527 (100) |  | 4,265 (100) |  | 10,175 ( 75) |
|  |  | Excluded |  | 0 ( 0) |  | 0 ( 0) |  | 0 ( 0) |  | 0 ( 0) |
|  |  | Missing data |  | 1,642 ( 35) |  | 0 ( 0) |  | 0 ( 0) |  | 1,642 ( 12) |
|  |  |  |  |  |  |  |  |  |  |  |
|  |  |  |  |  |  |  |  |  |  |  |
| Abbreviations: NHANES = National Health and Nutrition Examination Survey. | | | | | | | | | | |
| ^a^ For certain factors, some participants in specific time periods and/or age groups were not surveyed by NHANES, some participants were excluded at the analysis stage so that all time periods would involve the same age groups, and some participants were missing data due to nonresponse or other reasons beyond our control. | | | | | | | | | | |
| ^b^ Data on alcohol consumption were not collected by NHANES for any participants under age 20 years. | | | | | | | | | | |
| ^c^ Data on C-reactive protein were not collected by NHANES for any participants in Period 3. | | | | | | | | | | |
| ^d^ Data on general health status were not collected by NHANES for any participants in Period 1 and the first cycle of Period 2 (1999-2000) or for participants under age 20 years in the second cycle of Period 2 (2001-2002). For consistency across periods, we excluded all participants under age 20 years when analyzing general health status. | | | | | | | | | | |
| ^e^ Data on poor health preventing work were not collected by NHANES for any participants in Period 1 or for participants under age 20 years in Periods 2 and 3. | | | | | | | | | | |
| ^f^ Allergens included Alternaria alternata, Bermuda grass, cat, German cockroach, dust mite, peanut, short ragweed, Russian thistle, rye grass, and white oak. Data on allergens were not collected by NHANES for any participants in Periods 2 and 3, and were collected only for participants under age 20 years and a random half-sample of participants age 20-59 years in Period 1. | | | | | | | | | | |

| Supplemental Table S3. Sample distributions of total ANA and anti-DFS70 antibody positivity in additional subgroups. | | | | | | | | |
| --- | --- | --- | --- | --- | --- | --- | --- | --- |
|  |  |  |  |  |  |  |  |  |
|  |  |  |  |  |  |  |  |  |
|  |  |  |  | Number |  | Number (%) of Participants Positive for: | | |
| Factor of Interest |  | Subgroup |  | Analyzed ^a^ |  | Total ANA |  | Anti-DFS70 |
|  |  |  |  |  |  |  |  |  |
| Education |  | Less than high school |  | 4,568 |  | 613 (13.4) |  | 78 (1.7) |
|  |  | High school/GED |  | 3,119 |  | 410 (13.2) |  | 68 (2.2) |
|  |  | More than high school |  | 5,445 |  | 770 (14.1) |  | 154 (2.8) |
|  |  |  |  |  |  |  |  |  |
| C-reactive Protein (mg/L) |  | Low/moderate (≤3) |  | 6,342 |  | 732 (11.5) |  | 123 (1.9) |
|  |  | High (>3 to 10) |  | 2,056 |  | 306 (14.9) |  | 45 (2.2) |
|  |  | Very high (>10) |  | 758 |  | 135 (17.8) |  | 10 (1.3) |
|  |  |  |  |  |  |  |  |  |
| General Health Status |  | Excellent |  | 613 |  | 85 (13.9) |  | 22 (3.6) |
| (age ≥20 years) |  | Very good |  | 1,473 |  | 202 (13.7) |  | 48 (3.3) |
|  |  | Good |  | 2,096 |  | 323 (15.4) |  | 52 (2.5) |
|  |  | Fair/poor |  | 1,187 |  | 212 (17.9) |  | 38 (3.2) |
|  |  |  |  |  |  |  |  |  |
| Poor Health Prevents Work |  | No |  | 6,006 |  | 866 (14.4) |  | 170 (2.8) |
| (age ≥20 years) |  | Yes |  | 920 |  | 159 (17.3) |  | 18 (2.0) |
|  |  |  |  |  |  |  |  |  |
| Positive for Allergens ^b^ |  | No |  | 751 |  | 65 ( 8.7) |  | 10 (1.3) |
| (age <60 years) |  | Yes |  | 951 |  | 85 ( 8.9) |  | 15 (1.6) |
|  |  |  |  |  |  |  |  |  |
|  |  |  |  |  |  |  |  |  |
| Abbreviations: ANA = antinuclear antibodies; Anti-DFS70 = subclass of ANA that had the DFS staining pattern and bound the 70 kDa DFS protein in an enzyme-linked immunosorbent assay; DFS = dense fine speckled. | | | | | | | | |
| ^a^ The subgroup counts do not sum to the overall total of 13,519 because some participants were not surveyed, were excluded, or were missing data (see Supplemental Table S2 for details and exact counts). | | | | | | | | |
| ^b^ Allergens included Alternaria alternata, Bermuda grass, cat, German cockroach, dust mite, peanut, short ragweed, Russian thistle, rye grass, and white oak. | | | | | | | | |

| Supplemental Table S4. Prevalence estimates for total ANA and anti-DFS70 antibody positivity in additional subgroups. | | | | | | |
| --- | --- | --- | --- | --- | --- | --- |
|  |  |  |  |  |  |  |
|  |  |  |  |  |  |  |
|  |  |  |  | Prevalence Estimate (95% CI) of Positivity for: ^a^ | | |
| Factor of Interest |  | Subgroup |  | Total ANA |  | Anti-DFS70 |
|  |  |  |  |  |  |  |
| Education |  | Less than high school |  | 12.4 (10.9, 14.0) |  | 2.08 (1.35, 3.20) |
|  |  | High school/GED |  | 11.7 (10.1, 13.5) |  | 2.32 (1.69, 3.17) |
|  |  | More than high school |  | 13.6 (12.3, 15.1) |  | 3.20 (2.53, 4.03) |
|  |  |  |  |  |  |  |
| C-reactive Protein (mg/L) |  | Low/moderate (≤3) |  | 10.2 ( 9.2, 11.3) |  | 2.05 (1.59, 2.64) |
|  |  | High (>3 to 10) |  | 12.7 (10.9, 14.8) |  | 2.28 (1.54, 3.37) |
|  |  | Very high (>10) |  | 17.0 (13.3, 21.6) |  | 1.60 (0.69, 3.68) |
|  |  |  |  |  |  |  |
| General Health Status |  | Excellent |  | 15.4 (11.7, 19.9) |  | 4.51 (2.70, 7.46) |
| (age ≥20 years) |  | Very good |  | 13.3 (11.5, 15.4) |  | 3.95 (2.83, 5.49) |
|  |  | Good |  | 15.8 (13.7, 18.1) |  | 3.43 (2.40, 4.87) |
|  |  | Fair/poor |  | 16.9 (14.3, 19.8) |  | 3.51 (2.28, 5.36) |
|  |  |  |  |  |  |  |
| Poor Health Prevents Work |  | No |  | 14.2 (12.9, 15.6) |  | 3.54 (2.83, 4.41) |
| (age ≥20 years) |  | Yes |  | 14.9 (12.7, 17.4) |  | 2.04 (1.14, 3.63) |
|  |  |  |  |  |  |  |
| Positive for Allergens ^b^ |  | No |  | 7.3 ( 4.4, 11.7) |  | 0.47 (0.17, 1.28) |
| (age <60 years) |  | Yes |  | 7.2 ( 5.3, 9.6) |  | 0.75 (0.36, 1.56) |
|  |  |  |  |  |  |  |
|  |  |  |  |  |  |  |
| Abbreviations: ANA = antinuclear antibodies; Anti-DFS70 = subclass of ANA that had the DFS staining pattern and bound the 70 kDa DFS protein in an enzyme-linked immunosorbent assay; CI = confidence interval; DFS = dense fine speckled. | | | | | | |
| ^a^ Prevalence was estimated under a logistic regression model for total ANA or anti-DFS70 antibody positivity and expressed as the percent positive. Each model adjusted for the survey design variables (sampling strata, clusters, and weights) and included a categorical covariate for the factor of interest. | | | | | | |
| ^b^ Allergens included Alternaria alternata, Bermuda grass, cat, German cockroach, dust mite, peanut, short ragweed, Russian thistle, rye grass, and white oak. | | | | | | |

| Supplemental Table S5. Covariate-adjusted odds ratios for assessing associations between additional factors and total ANA, anti-DFS70 antibodies, and other ANA. | | | | | | | | |
| --- | --- | --- | --- | --- | --- | --- | --- | --- |
|  |  |  |  |  |  |  |  |  |
|  |  |  |  |  |  |  |  |  |
|  |  |  |  | P-value or Odds Ratio (95% CI) of Positivity for: ^a^ | | | | |
| Factor of Interest |  | Category |  | Total ANA |  | Anti-DFS70 |  | Other ANA |
|  |  |  |  |  |  |  |  |  |
| Education |  | Less than high school |  | P=0.070 |  | P=0.529 |  | P=0.235 |
|  |  | High school/GED |  | 1.04 (0.84, 1.29) |  | 1.10 (0.62, 1.96) |  | 1.03 (0.81, 1.31) |
|  |  | More than high school |  | 1.22 (1.03, 1.46) |  | 1.28 (0.78, 2.11) |  | 1.21 (0.95, 1.54) |
|  |  |  |  |  |  |  |  |  |
| C-reactive Protein (mg/L) |  | Low/moderate (≤3) |  | P=0.360 |  | P=0.376 |  | P=0.115 |
|  |  | High (>3 to 10) |  | 1.04 (0.84, 1.29) |  | 0.94 (0.59, 1.52) |  | 1.06 (0.84, 1.34) |
|  |  | Very high (>10) |  | 1.25 (0.92, 1.70) |  | 0.56 (0.25, 1.27) |  | 1.42 (1.02, 1.96) |
|  |  |  |  |  |  |  |  |  |
| General Health Status |  | Excellent |  | P=0.182 |  | P=0.863 |  | P=0.111 |
| (age ≥20 years) |  | Very good |  | 0.82 (0.56, 1.19) |  | 0.86 (0.46, 1.61) |  | 0.80 (0.52, 1.24) |
|  |  | Good |  | 0.97 (0.71, 1.33) |  | 0.79 (0.44, 1.41) |  | 1.04 (0.70, 1.55) |
|  |  | Fair/poor |  | 0.99 (0.65, 1.50) |  | 0.87 (0.39, 1.94) |  | 1.03 (0.66, 1.61) |
|  |  |  |  |  |  |  |  |  |
| Poor Health Prevents Work |  | No |  | P=0.259 |  | P=0.104 |  | P=0.738 |
| (age ≥20 years) |  | Yes |  | 0.88 (0.70, 1.10) |  | 0.58 (0.30, 1.12) |  | 0.96 (0.75, 1.23) |
|  |  |  |  |  |  |  |  |  |
| Positive for Allergens ^b^ |  | No |  | P=0.531 |  | P=0.349 |  | P=0.673 |
| (age <60 years) |  | Yes |  | 1.18 (0.70, 1.99) |  | 2.07 (0.43, 9.96) |  | 1.11 (0.67, 1.85) |
|  |  |  |  |  |  |  |  |  |
|  |  |  |  |  |  |  |  |  |
| Abbreviations: ANA = antinuclear antibodies; Anti-DFS70 = subclass of ANA that had the DFS staining pattern and bound the 70 kDa DFS protein in an enzyme-linked immunosorbent assay; CI = confidence interval; DFS = dense fine speckled; Other ANA = all types of ANA except anti-DFS70 antibodies. | | | | | | | | |
| ^a^ Each association was assessed by estimating an odds ratio under a logistic regression model for the prevalence of Total ANA, Anti-DFS70, or Other ANA. The model adjusted for the survey design variables (sampling strata, clusters, and weights), a restricted cubic spline in age, and categorical covariates for time period, sex, race/ethnicity, and the factor of interest. The first category of each factor is the referent; thus, its odds ratio is 1.00 by definition and is not shown. Instead, we show a P-value that indicates the statistical significance of the association between the factor as a whole and antibody status, based on an F-test from a statistical contrast. | | | | | | | | |
| ^b^ Allergens included Alternaria alternata, Bermuda grass, cat, German cockroach, dust mite, peanut, short ragweed, Russian thistle, rye grass, and white oak. | | | | | | | | |

| Supplemental Table S6. Covariate-adjusted assessments of time trends for total ANA, anti-DFS70 antibodies, and other ANA in additional subgroups.^a^ | | | | | | | | | | | | |
| --- | --- | --- | --- | --- | --- | --- | --- | --- | --- | --- | --- | --- |
|  |  |  |  |  |  |  |  |  |  |  |  |  |
|  |  |  |  |  |  | Prevalence Odds Ratio (95% CI) for Time Period | | | | |  | Trend |
| Factor of Interest |  | Subgroup |  | Response |  | 1988-1991 |  | 1999-2004 |  | 2011-2012 |  | P-value |
|  |  |  |  |  |  |  |  |  |  |  |  |  |
| Education |  | Less than high |  | Total ANA |  | 1.00 |  | 0.96 (0.69, 1.35) |  | 1.35 (0.93, 1.95) |  | 0.143 |
|  |  | school |  | Anti-DFS70 |  | 1.00 |  | 1.40 (0.61, 3.21) |  | 1.98 (0.72, 5.46) |  | 0.191 |
|  |  |  |  | Other ANA |  | 1.00 |  | 0.91 (0.61, 1.35) |  | 1.24 (0.79, 1.93) |  | 0.404 |
|  |  |  |  |  |  |  |  |  |  |  |  |  |
|  |  | High school |  | Total ANA |  | 1.00 |  | 0.93 (0.63, 1.38) |  | 1.23 (0.82, 1.84) |  | 0.388 |
|  |  | or GED |  | Anti-DFS70 |  | 1.00 |  | 2.68 (1.16, 6.19) |  | 2.57 (1.05, 6.28) |  | 0.020 |
|  |  |  |  | Other ANA |  | 1.00 |  | 0.73 (0.48, 1.09) |  | 1.04 (0.70, 1.54) |  | 0.957 |
|  |  |  |  |  |  |  |  |  |  |  |  |  |
|  |  | More than high |  | Total ANA |  | 1.00 |  | 1.04 (0.80, 1.36) |  | 1.59 (1.21, 2.09) |  | 0.001 |
|  |  | school |  | Anti-DFS70 |  | 1.00 |  | 1.31 (0.60, 2.84) |  | 3.11 (1.60, 6.03) |  | 0.001 |
|  |  |  |  | Other ANA |  | 1.00 |  | 0.99 (0.75, 1.32) |  | 1.30 (0.97, 1.73) |  | 0.061 |
|  |  |  |  |  |  |  |  |  |  |  |  |  |
| C-reactive Protein |  | Low to |  | Total ANA |  | 1.00 |  | 1.06 (0.84, 1.33) |  | ----- |  | 0.639 |
| (mg/L) |  | Moderate (≤3) |  | Anti-DFS70 |  | 1.00 |  | 1.51 (0.91, 2.52) |  | ----- |  | 0.111 |
|  |  |  |  | Other ANA |  | 1.00 |  | 0.97 (0.76, 1.23) |  | ----- |  | 0.777 |
|  |  |  |  |  |  |  |  |  |  |  |  |  |
|  |  | High (>3 to 10) |  | Total ANA |  | 1.00 |  | 0.74 (0.50, 1.09) |  | ----- |  | 0.125 |
|  |  |  |  | Anti-DFS70 |  | 1.00 |  | 1.39 (0.63, 3.09) |  | ----- |  | 0.411 |
|  |  |  |  | Other ANA |  | 1.00 |  | 0.65 (0.43, 0.97) |  | ----- |  | 0.034 |
|  |  |  |  |  |  |  |  |  |  |  |  |  |
|  |  | Very high (>10) |  | Total ANA |  | 1.00 |  | 1.49 (0.79, 2.82) |  | ----- |  | 0.215 |
|  |  |  |  | Anti-DFS70 |  | 1.00 |  | 31.6 (6.61, 151) |  | ----- |  | <0.0001 |
|  |  |  |  | Other ANA |  | 1.00 |  | 1.29 (0.69, 2.44) |  | ----- |  | 0.423 |
|  |  |  |  |  |  |  |  |  |  |  |  |  |
| General Health |  | Excellent |  | Total ANA |  | ----- |  | 1.00 |  | 2.18 (1.14, 4.16) |  | 0.018 |
| Status |  |  |  | Anti-DFS70 |  | ----- |  | 1.00 |  | 1.55 (0.51, 4.76) |  | 0.436 |
| (age ≥20 years) |  |  |  | Other ANA |  | ----- |  | 1.00 |  | 2.41 (1.20, 4.87) |  | 0.014 |
|  |  |  |  |  |  |  |  |  |  |  |  |  |
|  |  | Very good |  | Total ANA |  | ----- |  | 1.00 |  | 1.47 (1.02, 2.12) |  | 0.037 |
|  |  |  |  | Anti-DFS70 |  | ----- |  | 1.00 |  | 1.95 (0.86, 4.42) |  | 0.106 |
|  |  |  |  | Other ANA |  | ----- |  | 1.00 |  | 1.31 (0.89, 1.93) |  | 0.169 |
|  |  |  |  |  |  |  |  |  |  |  |  |  |
|  |  | Good |  | Total ANA |  | ----- |  | 1.00 |  | 1.42 (0.98, 2.05) |  | 0.062 |
|  |  |  |  | Anti-DFS70 |  | ----- |  | 1.00 |  | 1.78 (0.85, 3.73) |  | 0.121 |
|  |  |  |  | Other ANA |  | ----- |  | 1.00 |  | 1.31 (0.90, 1.93) |  | 0.156 |
|  |  |  |  |  |  |  |  |  |  |  |  |  |
|  |  | Fair to Poor |  | Total ANA |  | ----- |  | 1.00 |  | 1.24 (0.83, 1.88) |  | 0.290 |
|  |  |  |  | Anti-DFS70 |  | ----- |  | 1.00 |  | 0.62 (0.25, 1.52) |  | 0.286 |
|  |  |  |  | Other ANA |  | ----- |  | 1.00 |  | 1.47 (0.92, 2.34) |  | 0.106 |
|  |  |  |  |  |  |  |  |  |  |  |  |  |
| Poor Health |  | No |  | Total ANA |  | ----- |  | 1.00 |  | 1.45 (1.16, 1.81) |  | 0.001 |
| Prevents Work |  |  |  | Anti-DFS70 |  | ----- |  | 1.00 |  | 1.72 (1.10, 2.69) |  | 0.018 |
| (age ≥20 years) |  |  |  | Other ANA |  | ----- |  | 1.00 |  | 1.35 (1.11, 1.65) |  | 0.003 |
|  |  |  |  |  |  |  |  |  |  |  |  |  |
|  |  | Yes |  | Total ANA |  | ----- |  | 1.00 |  | 1.94 (1.24, 3.03) |  | 0.004 |
|  |  |  |  | Anti-DFS70 |  | ----- |  | 1.00 |  | 1.71 (0.50, 5.88) |  | 0.390 |
|  |  |  |  | Other ANA |  | ----- |  | 1.00 |  | 1.95 (1.21, 3.16) |  | 0.007 |
|  |  |  |  |  |  |  |  |  |  |  |  |  |
|  |  |  |  |  |  |  |  |  |  |  |  |  |
| Abbreviations: ANA = antinuclear antibodies; Anti-DFS70 = subclass of ANA that had the DFS staining pattern and bound the 70 kDa DFS protein in an enzyme-linked immunosorbent assay; CI = confidence interval; DFS = dense fine speckled; Other ANA = all types of ANA except anti-DFS70 antibodies. | | | | | | | | | | | | |
| ^a^ Time trend assessments were based on two logistic regression models for antibody positivity, which adjusted for the survey design variables (sampling strata, clusters, and weights), a restricted cubic spline in age, and categorical covariates for sex and race/ethnicity. The first model added a categorical covariate for time period and estimated the prevalence odds ratio for each period, relative to the earliest period with data. The second model added a quantitative covariate for the number of years between period midpoints, relative to the earliest period with data, and produced a P-value from a χ^2^-test to assess a time trend. | | | | | | | | | | | | |

| Supplemental Table S7. Covariate-adjusted tests of whether time trends differed across factor-specific subgroups. ^a^ | | | | |
| --- | --- | --- | --- | --- |
|  |  |  |  |  |
|  |  |  |  |  |
|  |  | P-value for Test of Equal Time Trends Across Subgroups | | |
| Factor of Interest |  | Total ANA |  | Anti-DFS70 |
|  |  |  |  |  |
| Sex |  | 0.068 |  | 0.025 |
|  |  |  |  |  |
| Age (years) |  | 0.036 |  | 0.911 |
|  |  |  |  |  |
| Race/Ethnicity |  | 0.002 |  | 0.150 |
|  |  |  |  |  |
| Body Mass Index (BMI) |  | 0.060 |  | 0.167 |
|  |  |  |  |  |
| Poverty Income Ratio (PIR) |  | 0.349 |  | 0.005 |
|  |  |  |  |  |
| Current Smoking Exposure (ng/ml of cotinine) |  | 0.904 |  | 0.034 |
|  |  |  |  |  |
| Alcohol Consumption (ages ≥20 years) |  | 0.014 |  | 0.112 |
|  |  |  |  |  |
| Education |  | 0.599 |  | 0.530 |
|  |  |  |  |  |
| C-reactive Protein (mg/L) |  | 0.211 |  | 0.002 |
|  |  |  |  |  |
| General Health Status (ages ≥20 years) |  | 0.923 |  | 0.271 |
|  |  |  |  |  |
| Poor Health Prevents Work (ages ≥20 years) |  | 0.230 |  | 0.915 |
|  |  |  |  |  |
|  |  |  |  |  |
| Abbreviations: ANA = antinuclear antibodies; Anti-DFS70 = subclass of ANA that had the DFS staining pattern and bound the 70 kDa DFS protein in an enzyme-linked immunosorbent assay; DFS = dense fine speckled. | | | | |
| ^a^ The null hypothesis of identical time trends in all subgroups defined by the categories of a given factor was evaluated with an F-test from a statistical contrast. A logistic regression model was assumed for either Total ANA or Anti-DFS70. The model adjusted for the survey design variables (sampling strata, clusters, and weights), a restricted cubic spline in age, categorical covariates for sex and race/ethnicity, a quantitative covariate for time (the number of years between period midpoints, relative to the earliest period with data), and an interaction between time and the factor of interest. When age was the factor of interest, a categorical covariate was substituted for the cubic spline. The reported P-value indicates the statistical significance when testing the interaction coefficient(s) equaled zero. | | | | |
